# Online peer support for breast cancer survivors: protocol for a decentralized multicenter open-label pilot randomized controlled trial (HOPE-BC study)

**DOI:** 10.1007/s10147-026-02979-3

**Published:** 2026-02-13

**Authors:** Ken Kurisu, Yosuke Uchitomi, Naomi Sakurai, Maiko Fujimori, Nobuya Akizuki, Asao Ogawa, Akifumi Kurata, Tadahiro Izutani, Keita Mori, Tatsuo Akechi, Toshinari Yamashita

**Affiliations:** 1 Department of Cancer Survivorship and Digital Medicine, The Jikei University School of Medicine, Tokyo, Japan; 2 Cancer Solutions Inc., Tokyo, Japan; 3 Division of Survivorship Research, National Cancer Center Institute for Cancer Control, Tokyo, Japan; 4 Department of Psycho-Oncology, Tokyo Metropolitan Cancer and Infectious Diseases Center Komagome Hospital, Tokyo, Japan; 5 Division of Psycho-Oncology, Exploratory Oncology Research and Clinical Trial Center, National Cancer Center, Kashiwa, Chiba Japan; 6 Daiichi Sankyo Co., Ltd., Tokyo, Japan; 7 Department of Biostatistics, Shizuoka Cancer Center, Shizuoka, Japan; 8 Department of Psychiatry and Cognitive-Behavioral Medicine, Graduate School of Medical Sciences, Nagoya City University, Nagoya, Japan; 9 Department of Breast Surgery and Oncology, Kanagawa Cancer Center, Yokohama, Japan

**Keywords:** Peer support, Breast cancer survivors, Randomized controlled trial, Decentralized clinical trial, Matching algorithm

## Abstract

**Background:**

The effectiveness of peer support for breast cancer survivors has varied substantially, and no previous study has examined patient–supporter matching. This study protocol describes a decentralized, multicenter, open-label pilot randomized controlled trial designed to explore the effectiveness of online peer support for breast cancer survivors and to develop a matching algorithm for use in future research.

**Methods:**

Fifty breast cancer survivors within 3 years of completing initial treatment will be recruited and will provide electronic informed consent. They will be randomly assigned to either the peer support group, which will schedule a peer support session immediately after registration, or the waitlist control group, which will schedule their session 2 weeks later. Peer support sessions will be conducted online by two trained peer supporters. The primary outcome is the score on the UCLA Loneliness Scale, assessed 1 week after the session in the peer support group and at the end of the 2-week waiting period in the control group. Secondary outcomes include satisfaction with peer support and other psychosocial measures, all assessed through an electronic patient-reported outcome system. Effect sizes will be calculated to inform sample size estimation for future trials. Potential factors associated with satisfaction, such as similarity between participants and peer supporters, will also be explored to guide matching algorithm development.

**Discussion:**

The findings will inform a future confirmatory trial incorporating an optimized matching algorithm.

*Trial registration* UMIN000056741.

**Supplementary Information:**

The online version contains supplementary material available at 10.1007/s10147-026-02979-3.

## Introduction

The incidence of breast cancer in Japan is increasing, and advances in treatment have improved the 10-year survival to nearly 90% [1]. Cases begin to rise among individuals in their late 30s and peak among those in their 40–60s [1], requiring many patients to manage both treatment and daily responsibilities at work and home [2]. Peer support, in which cancer survivors share their experiences with newly diagnosed individuals, has been used increasingly as a supportive resource for patients and families. Since 2018, the Ministry of Health, Labour and Welfare, in partnership with the Japanese Psycho-Oncology Society, has promoted peer supporter training by developing training manuals and conducting workshops [3]. Social participation and support alleviate loneliness among cancer survivors, thereby improving depressive mood and quality of life, especially in younger survivors [4].

However, a systematic review has shown substantial heterogeneity in the effects of peer support for individuals with breast cancer [5]. Peer support is not recommended for alleviating psychological distress in relevant clinical guidelines [6, 7]. One possible explanation for heterogeneity in the effectiveness of peer support and the eventual limited evidence may stem from inadequate optimization of peer supporter matching. Modern web and smartphone applications incorporate data-driven optimization to enhance service quality [8]. In non-breast cancer populations, studies have examined optimized matching in peer support and identified several factors related to the effectiveness of peer support [9–11]. Nevertheless, in Japan, peer support is typically offered through in-person hospital-based programs, where the supporter available at the moment responds to the patient. Consequently, they may not necessarily receive peer support relevant to their topics of interest. Furthermore, while loneliness is a crucial psychological factor in younger cancer survivors [4], previous studies of peer support have inadequately examined loneliness [5].

Therefore, optimized matching may enhance the effectiveness of online peer support. This approach may complement existing medical resources and help reduce regional disparities in Japan, where psycho-oncology specialists are scarce [12], and may also enhance peer supporters’ self-efficacy through engagement. This pilot randomized controlled trial (Helping each Other by Peer Empowerment for Breast Cancer [HOPE-BC]) aims to generate preliminary data on effect sizes, particularly regarding loneliness outcomes, for future research. A second aim is to examine factors influencing participants’ satisfaction with peer support to help develop a matching algorithm for use in future research.

## Methods

### Ethics approval

This study protocol (version 1.1; May 27, 2025) was approved by the Institutional Review Board of the non-profit organization MINS (IRB No: DS-HaaS-005). Informed consent will be obtained from all participants. The study has been registered in a publicly accessible database (University Hospital Medical Information Network Clinical Trials Registry [UMIN-CTR]; registration number: UMIN000056741; registered on 2025 Jan 17).

### Study design

This study is a multicenter, non-invasive, open-label, pilot randomized controlled trial. The following nine medical institutions will participate: Kanagawa Cancer Center, Hokkaido University Hospital, Hachinohe City Hospital, Akita University Hospital, Jikei University Hospital, Nagoya City University Hospital, Otani Shoichiro Breast Clinic, Kumamoto University Hospital, and Naha-Nishi Clinic. This protocol adheres to the SPIRIT 2013 guidelines and the CONSORT extension for pilot and feasibility trials (see Supplementary Materials for the checklists and planned flow diagram).

### Participants

In this study, peer support will focus on psychosocial problems following the completion of physical treatment. Thus, this study will enroll individuals with early-stage breast cancer who have completed initial treatment.

The eligibility criteria are: (1) age 18 years or older; (2) female; (3) diagnosis of early-stage breast cancer; (4) enrollment within 3 years after completion of initial treatment for breast cancer (participants scheduled for surgery may be enrolled postoperatively; those scheduled for adjuvant therapy may be enrolled after completion or discontinuation of the planned adjuvant therapy; however, those receiving adjuvant endocrine therapy alone may be enrolled even before completion or discontinuation of such therapy); (5) daily use of digital devices and ability to participate in online peer support; and (6) provision of informed consent via an electronic system.

The exclusion criteria are: (1) active concurrent cancers, except for ductal carcinoma in situ; and (2) ineligibility for any reason determined by the attending physician or investigator.

### Procedure

**Fig. 1:**
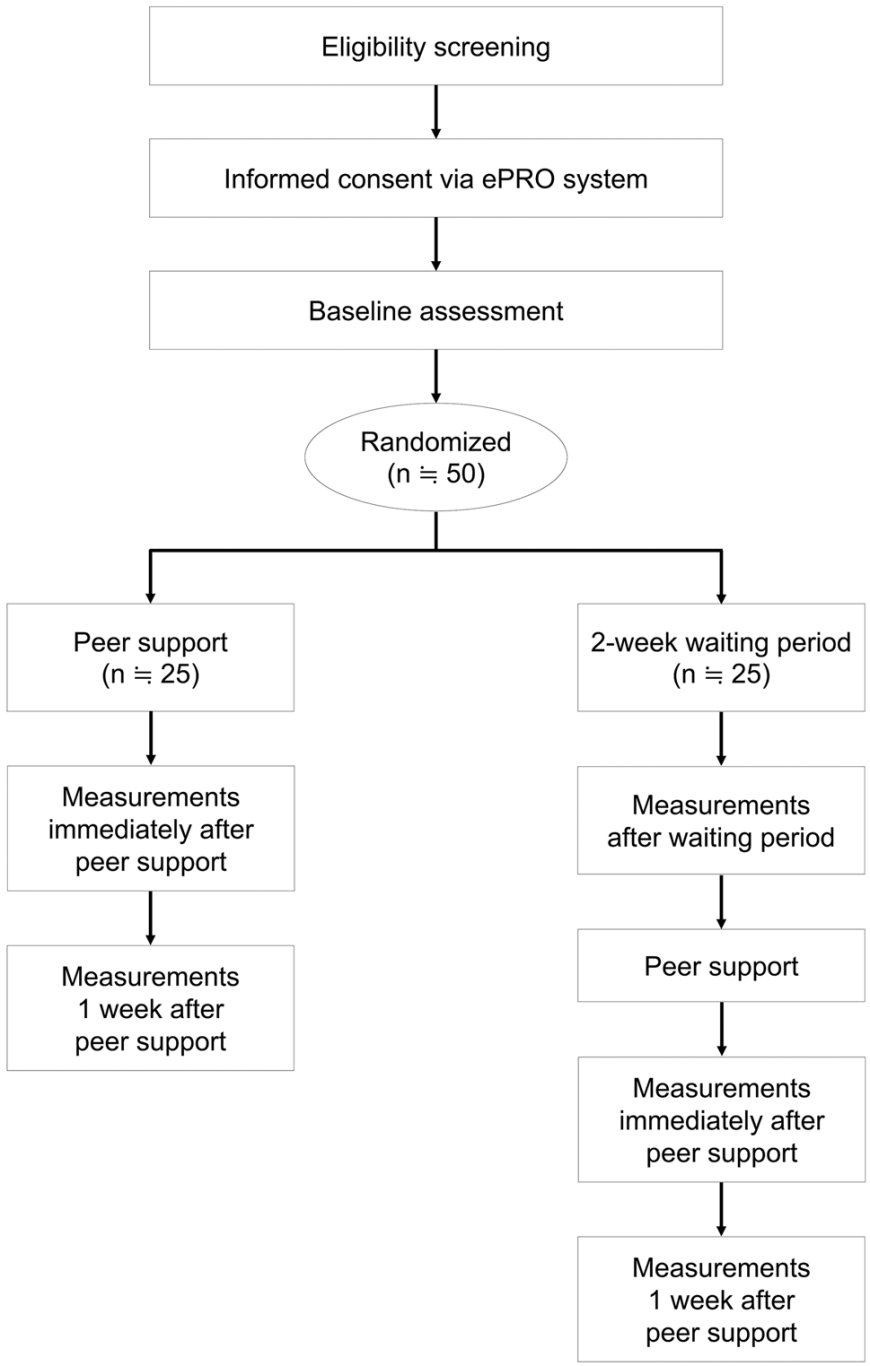
Flow of participants through the study

Figure 1 shows the flowchart of the study procedure. This study will conduct a decentralized clinical trial (DCT) [13–17]. Physicians at participating institutions will identify eligible individuals during routine clinical practice and provide study information via a leaflet containing a QR code. The candidates will scan the QR code using their own digital device, view an explanatory video, review the study details, and provide electronic informed consent. Eligibility will then be confirmed by the study coordinating team via telephone, and identity will be verified by uploading a medical ID card image.

Enrolled participants will access the registration page and enter demographic, clinical, and psychosocial information, and topics of interest selected from 18 predefined items (Supplementary Table 1). These items were developed based on the Zenganren × J-SUPPORT × SaQRA Research and Development Map [18].

Using Hibilog, an Interactive Web Response System (Accelight, Inc.), participants will be randomized using permuted block randomization to either the peer support group, which will immediately schedule a session, or the waitlist control group, which will schedule a session 2 weeks after registration. The block size will be confidential, with access limited to the system administrator. No stratification factors will be applied, given the small sample size in this pilot study. This randomized design with a waitlist group will enable us to simultaneously estimate intervention effects and examine satisfaction with the peer support session using data from both groups.

Participants will then attend peer support sessions and complete a suite of outcome measures described below. They may receive up to 4,000 Japanese yen (approximately 28 USD based on the exchange rate at the time of study planning) in Amazon gift cards based on completion of study questionnaires.

### Peer support

Participants randomized to the peer support group will schedule a session immediately after registration, while those in the waitlist group will schedule their session 2 weeks later. Reminders will be sent as needed by the study coordinating team if no reservation is made. Appointments will be made via a web system, allowing participants to select preferred dates and times from slots pre-specified by peer supporters. Participants in the waitlist group will be instructed not to engage in other peer support activities during the waiting period.

Each participant will receive a single approximately 40-min online session via Zoom, a web-based video conferencing system (Zoom Video Communications, Inc.). The session will be conducted by two peer supporters. Peer supporters will be provided with a manual that describes the procedures and general guidance, and the outline is shown in Supplementary Table 2. A primary supporter will lead the session, and a secondary supporter will manage time, record the session, and request questionnaire completion at the end. The second supporter will reduce the burden on the primary supporter by handling these tasks and will enhance session quality by addressing participants’ questions that the primary supporter may not be able to answer adequately. Peer supporters will report any issues occurring during each session, including technical problems (e.g., connectivity issues), adherence to session duration, and other difficulties encountered during the session.

Peer supporters will be recruited by CancerNet Japan, a non-profit organization that provides evidence-based cancer information and has offered a training program for breast cancer survivors to become peer supporters since 2007 [19]. Most graduates of this course engage in patient group activities or peer support, and CancerNet Japan maintains a nationwide network.

The eligibility criteria for peer supporters are: (1) female breast cancer survivors; (2) completion of the training program developed by the Ministry of Health, Labour and Welfare [3]; and (3) prior peer support experience. Criteria (2) and (3) are intended to ensure session quality. The training program is designed for individuals interested in peer support and focuses on sharing personal experiences with others [3]. It also provides guidance on protecting privacy and maintaining appropriate boundaries, such as refraining from offering opinions or advice regarding diagnoses or treatment.

At study initiation, peer supporters will report their demographic and social characteristics, indicate topics they have experienced, and complete the Japanese version of the Posttraumatic Growth Inventory (PTGI-J) [20].

### Measurements

Participants will complete web-based questionnaires via an electronic patient-reported outcome (ePRO) system at the following time points: registration, the end of the 2-week waiting period (for the waitlist group only), immediately after the peer support session, and 1 week after the session (Table 1). For the assessments at the end of the waiting period and 1 week after the peer support session, a 1-week window will be allowed, with automated reminders sent by the system if no response is recorded.

**Table 1:** Measurement schedule

	Baseline	End of waiting period^a^^,b^	Immediately after peer support	1 week after peer support^b^
UCLA loneliness scale	●	●		●
Satisfaction			●	●
Usefulness			●	●
GAD-7	●	●		●
PHQ-9	●	●		●
CARS-J	●	●		●
SCNS-SF34-J	●	●		●
mDAMS	●	●	●	●
EORTC QLQ-C30	●	●		●

^a^Waitlist group only

^b^One-week window allowed

*GAD-7*, generalized anxiety disorder-7; *PHQ-9*, patient health questionnaire-9; *CARS-J*, concerns about recurrence scale Japanese version; *SCNS-SF34*, supportive care needs survey short form-34; *mDAMS*, modified version of the depression and anxiety mood scale; *EORTC QLQ-C30*, European Organization for Research and Treatment of Cancer quality of life questionnaire core 30

The primary outcome is the effect size of the intervention to reduce loneliness level measured using the UCLA Loneliness Scale 1 week after the peer support session. This 20-item questionnaire, each scored on a 1–4 point scale and a total score ranging from 20 to 80 points, assesses loneliness and has been validated in the Japanese population [21, 22].

Satisfaction with peer support will be used as an indicator for developing a matching algorithm. Items from the Japanese version of the Patient Satisfaction Questionnaire (PSQ-J) [23], which were originally used to assess satisfaction with medical care, are modified to assess satisfaction with peer support. The modified scale includes five items, each rated from 1 to 10 points, yielding a total score of 5–50 points.

To evaluate the usefulness of peer support, we developed an original ten-item measure consisting of nine items, each rated on a 7–point scale, and one open-ended comment for the session (Supplementary Table 3).

To measure anxiety over the past 2 weeks, the Generalized Anxiety Disorder-7 (GAD-7) will be used [24]. This consists of seven items plus one supplementary item, with total scores ranging from 0 to 21 points.

To measure depression over the past week, the Japanese version of the Patient Health Questionnaire-9 (PHQ-9) will be used [25, 26]. This is a nine-item questionnaire assessing depression severity, with scores ranging from 0 to 27 points.

The Concerns About Recurrence Scale Japanese version (CARS-J) will be used to assess fear of recurrence. This was developed for Japanese women with breast cancer and contains four items, each rated on a 6-point scale, and the total score ranges from 4 to 24 points [27].

The Supportive Care Needs Survey Short Form-34 (SCNS-SF34) measures supportive care needs across five domains [28]. This includes 34 items, each rated on a 1–5 point scale, with a total score ranging from 34 to 170 points.

Momentary mood will be assessed using a modified version of the Depression and Anxiety Mood Scale (mDAMS). We modified the items to measure current mood, whereas the original version assesses mood over the past few days [29]. The mDAMS includes nine items, each rated from 1 to 10 points. The scores on three items each are summed to yield positive mood, depressed mood, and anxiety scores.

Quality of life will be measured using the European Organization for Research and Treatment of Cancer Quality of Life Questionnaire Core 30 (EORTC QLQ-C30) [30, 31], a validated 30-item questionnaire. Items are rated on 1–4 or 1–7 point scales and converted to standardized scores ranging from 0 to 100 points for functional scales, symptom scales, and the global health status/quality of life scale.

### Statistical analyses

We will calculate effect sizes using mean differences and standard deviations, and compare UCLA Loneliness Scale scores between the peer support group at 1 week after the session and the waitlist group at the end of the 2-week waiting period. Comparisons will be performed using the Wilcoxon rank-sum test. A t-test will also be conducted when the score distributions approximate normality. The analysis will include all participants with available outcome data for comparison. The same analyses will be applied to other measures.

In addition, we will explore a linear regression model with participants’ satisfaction as the outcome. Explanatory variables will include participant characteristics, peer supporter characteristics, and similarity between participants and peer supporters (e.g., age difference, shared topics of interest). Data from all participants who complete satisfaction assessments after peer support will be included. This exploratory model will be refined, guided by the statistical significance of variables and the Akaike information criterion. Based on the final model, we will propose a potential approach for matching participants with peer supporters.

### Sample size calculation

One aim of this pilot study is to estimate the effect size required for a future randomized controlled trial, since no reference value is available in this study design. Therefore, we estimated the sample size assuming an effect size (Cohen’s d) of 0.3. A two-sample t-test with this effect size, 90% power, and a two-sided significance level of 5% requires 470 participants. Based on a previous study [32], we set the sample size at approximately 10% of that number, i.e., 50 participants.

In addition, a sample size of approximately 50 participants is considered sufficient for a multivariable linear regression model examining factors related to satisfaction with peer support. From this perspective as well, we concluded that 50 participants represent an appropriate sample size.

## Discussion

This pilot randomized controlled trial is designed to preliminarily explore the effect of online peer support for individuals with early-stage breast cancer. We will calculate effect sizes for each outcome and conduct preliminary analyses to develop a matching algorithm. We will then generate evidence for online peer support through a confirmatory trial that incorporates the matching strategy. Such an approach may provide psychosocial support without using medical resources and help address regional disparities in cancer care in Japan [12].

The strengths of this study will include the following. First, to our knowledge, no prior study has examined peer supporter matching for breast cancer survivors, and the findings will be novel. Second, the randomized design with the waitlist control group will enable us to simultaneously estimate intervention effects and explore a matching algorithm, even with a relatively small sample size. Third, the study will apply a DCT system [13–17], which will reduce the burden on both participants and clinicians substantially. Fourth, peer supporters will have prior experience and will have completed training provided by the Ministry of Health, Labour and Welfare [3]. While interventions by untrained peer supporters sometimes have adverse effects [5], such risk will be low in this study.

Nevertheless, several limitations should be noted. First, the intervention will include a single session, which may result in an insufficient effect size to achieve adequate statistical power. In addition, the effects of multiple sessions cannot be assessed in this trial. Future research may need to recruit a larger number of participants and examine the effectiveness of multiple sessions. Second, participants may receive informal support through social media or interactions at their medical institutions, which cannot be fully controlled or evaluated in this study. Third, although participants in the waitlist group will be instructed not to receive peer support outside the study during the waiting period, this protocol does not include procedures to monitor adherence to this instruction. In addition, psychosocial support outside the study will not be restricted due to ethical considerations. Therefore, monitoring and sensitivity analyses accounting for external exposure will be necessary in future studies. Fourth, while this protocol includes procedures to ensure and enhance the quality of peer support sessions, no quantitative scales to evaluate these aspects are included, which will need to be considered in future studies. Fifth, outcomes will be assessed 1 week after the session, and longer-term effects will remain unexplored. Sixth, given the many psychological measures collected, group differences may appear due to multiple testing. Thus, findings will need to be interpreted with caution. Finally, although this pilot study will provide insights into factors for matching, the small number of participants and peer supporters will limit the generalizability of these findings. Larger datasets will ultimately be required for model refinement. Taking these limitations into account, we will design subsequent confirmatory trials.

## Supplementary Information

Below is the link to the electronic supplementary material.

Supplementary material 1 (DOCX 43 KB)

Supplementary material 2 (DOCX 55 KB)

Supplementary material 3 (DOCX 62 KB)

Supplementary material 4 (DOCX 28 KB)

## Data Availability

No datasets were analyzed for this protocol, and there are no plans to make data publicly available.
