# Supplementary material for: Online peer support for breast cancer survivors: protocol for a decentralized multicenter open-label pilot randomized controlled trial (HOPE-BC study)
Source: Int J Clin Oncol. 2026 Feb 13;31(4):676–82. doi: 10.1007/s10147-026-02979-3 (PMC13018041; doi:10.1007/s10147-026-02979-3)
Supplement: Supplementary file 3 — Supplementary material 3 (DOCX 62 KB) [file 10147_2026_2979_MOESM3_ESM.docx]

Assessed for eligibility (n= )

Excluded (n= )

♦  Not meeting inclusion criteria (n= )

♦  Declined to participate (n= )

♦  Other reasons (n= )

Assessed for objective 1 (n= )

Assessed for objective 2 (n= ), Etc ...

Etc ..

Lost to follow-up (give reasons) (n= )

Discontinued intervention (give reasons) (n= )

Allocated to peer support group (n≒25)

♦ Received allocated intervention (n= )

♦ Did not receive allocated intervention (give reasons) (n= )

Lost to follow-up (give reasons) (n= )

Discontinued intervention (give reasons) (n= )

Allocated to waitlist control group (n≒25)

♦ Received allocated intervention (n= )

♦ Did not receive allocated intervention (give reasons) (n= )

Assessed for objective 1 (n= )

Assessed for objective 2 (n= )

## Allocation

## Assessment

## Follow-Up

Randomized (n≒50)

## Enrollment

Screened prior to eligibility assessment (n= )

Excluded (n= )

♦  Reasons (n= )

## Screened
