# Supplementary material for: Online peer support for breast cancer survivors: protocol for a decentralized multicenter open-label pilot randomized controlled trial (HOPE-BC study)
Source: Int J Clin Oncol. 2026 Feb 13;31(4):676–82. doi: 10.1007/s10147-026-02979-3 (PMC13018041; doi:10.1007/s10147-026-02979-3)
Supplement: Supplementary file 4 — Supplementary material 4 (DOCX 28 KB) [file 10147_2026_2979_MOESM4_ESM.docx]

**Supplementary Table 1. Topics of interest (translated from Japanese)**

| Topics |
| --- |
| 1. Emotional distress (including sleep problems) 2. Maintaining physical activity 3. Fatigue 4. Communication with the primary physician 5. Communication with family 6. Communication at the workplace 7. Nutrition and weight management 8. Accessing information 9. Postoperative pain 10. Early detection of recurrence and secondary prevention 11. Financial concerns 12. Employment 13. Lymphedema 14. Numbness 15. Constipation/diarrhea 16. Appearance (e.g., wigs) 17. Symptoms related to hormone therapy (e.g., menopausal disorders) 18. Others |

**Supplementary Table 2. Outline of the peer supporter manual**

| Header | Bullet points |
| --- | --- |
| Purpose and roles | - Primary supporter leads the session - Secondary supporter handles timekeeping, post-survey, and assists the primary supporter - Both cooperate to ensure high-quality support |
| General rules | - Avoid flashy clothing - Use a quiet and private space - Update Zoom to the latest version - Join 15 minutes early for quick coordination |
| Zoom setup | - Both Primary and Secondary keep camera ON - Use nicknames (avoid real names) - First entrant becomes host, and the second stays as co-host - Primary greets upon entry - Internal communication via chat |
| Before starting | - Participant must join from a private space - Short introductions - Participant may remain anonymous and off-camera - Duration around 40 min - No discussion of clinical decision-making - Technical issues may require termination |
| Conducting the Session | - Primary explains session flow and general rules - Free conversation after introductions - Review rules from training material by the Ministry of Health, Labour and Welfare - Take questions before starting |
| Troubleshooting | - If either supporter cannot join, cancel or proceed solo - If participant does not join, cancel after 10 min - Report any issues after the session |

**Supplementary Table 3. Questionnaire for usefulness of peer support (translated from Japanese)**

|  | Items | Categories |
| --- | --- | --- |
| 1 | Did the communication with the peer supporter help you understand your illness and treatment? | 1 = Not at all helpful  2 = Not helpful  3 = Somewhat not helpful  4 = Neutral  5 = Somewhat helpful  6 = Helpful  7 = Very helpful |
| 2 | Did the communication with the peer supporter help ease your mind? | Same as above |
| 3 | Did the communication with the peer supporter help improve your daily life? | Same as above |
| 4 | Overall, was the communication with the peer supporter helpful? | Same as above |
| 5 | Were you able to ask the questions you wanted during the peer support? | 1 = Not at all  2 = No  3 = Somewhat no  4 = Neutral  5 = Somewhat yes  6 = Yes  7 = Very much yes |
| 6 | Did you obtain hints about the concerns you had? | Same as above |
| 7 | Were the concerns you had beforehand resolved? | Same as above |
| 8 | Was it difficult to communicate with the peer supporter online instead of in person? | 1 = Very difficult  2 = Difficult  3 = Somewhat difficult  4 = Neutral  5 = Somewhat not difficult  6 = Not difficult  7 = Not difficult at all |
| 9 | Were you satisfied with online peer support instead of in person? | 1 = Not at all satisfied  2 = Not satisfied  3 = Somewhat not satisfied  4 = Neutral  5 = Somewhat satisfied  6 = Satisfied  7 = Very satisfied |
| 10 | Please write any other comments regarding peer support (free description).  *Please refrain from including comments that slander peer supporters or healthcare providers.* | Free description |
